# Captive Breeding and *Trichomonas gallinae* Alter the Oral Microbiome of Bonelli’s Eagle Chicks

**DOI:** 10.1007/s00248-022-02002-y

**Published:** 2022-04-07

**Authors:** Claudio Alba, José Sansano-Maestre, María Dolores Cid Vázquez, María del Carmen Martínez-Herrero, María Magdalena Garijo-Toledo, Iris Azami-Conesa, Virginia Moraleda Fernández, María Teresa Gómez-Muñoz, Juan Miguel Rodríguez

**Affiliations:** 1 Department of Nutrition and Food Science, Faculty of Veterinary Sciences, University Complutense of Madrid, Madrid, Spain; 2 Department of Animal Production and Public Health, Faculty of Veterinary and Experimental Sciences, Catholic University of Valencia, Valencia, Spain; 3 Department of Animal Health, Faculty of Veterinary Sciences, University Complutense of Madrid, Madrid, Spain; 4 Department of Animal Production and Health, Public Veterinary Health and Food Science and Technology, Faculty of Veterinary Medicine, Universidad Cardenal Herrera-CEU, CEU Universities, Valencia, Spain; 5 Grupo de Rehabilitación de la Fauna Autóctona y su Hábitat (GREFA), Madrid, Spain

**Keywords:** Oral microbiome, Raptors, *Gemella*, *Megamonas*, Bonelli’s eagle

## Abstract

Bonelli’s eagle (*Aquila fasciata*) is an endangered raptor species in Europe, and trichomonosis is one of the menaces affecting chicks at nest. In this paper, we attempt to describe the oral microbiome of Bonelli’s eagle nestlings and evaluate the influence of several factors, such as captivity breeding, *Trichomonas gallinae* infection, and the presence of lesions at the oropharynx. The core oral microbiome of Bonelli’s eagle is composed of Firmicutes, Bacteroidota, Fusobacteria and Proteobacteria as the most abundant phyla, and *Megamonas* and *Bacteroides* as the most abundant genera. None of the factors analysed showed a significant influence on alfa diversity, but beta diversity was affected for some of them. Captivity breeding exerted a high influence on the composition of the oral microbiome, with significant differences in the four most abundant phyla, with a relative increase of Proteobacteria and a decrease of the other three phyla in comparison with chicks bred at nest. Some genera were more abundant in captivity bred chicks, such as *Escherichia*-*Shigella*, *Enterococcus*, *Lactobacillus*, *Corynebacterium*, *Clostridium* and *Staphylococcus*, while *Bacteroides*, *Oceanivirga*, *Peptostreptococcus*, *Gemella*, *Veillonella*, *Mycoplasma*, *Suttonella*, *Alloscardovia*, *Varibaculum* and *Campylobacter* were more abundant in nest raised chicks*. T. gallinae* infection slightly influenced the composition of the microbiome, but chicks displaying trichomonosis lesions had a higher relative abundance of *Bacteroides* and *Gemella*, being the last one an opportunistic pathogen of abscess complications in humans. Raptor’s microbiomes are scarcely studied. This is the first study on the factors that influence the oral microbiome of Bonelli’s eagle.

**Supplementary Information:**

The online version contains supplementary material available at 10.1007/s00248-022-02002-y.

## Introduction

Bonelli’s eagle (*Aquila fasciata*) is an endangered species of raptor in Europe (920–1.100 couples) which is included in Annex I of the EU Directive 2009/147/EC of Birds and in Appendix II of CITES [1]. A dramatic decrease has been observed since 1959 in Europe [2]. Bonelli’s eagles extend along the Mediterranean basin, although 65% of the European population is in Spain. However, in some regions of the country, the population has declined up to 35% of the animals registered in the 1970s.

The main causes of mortality of adults and juveniles are collision with power lines, direct persecution by hunters, habitat changes and reduction of habitual preys [3]. Although corrective measures have been taken, including legislative actions, the regressive trend continues. Among the causes of mortality of chicks in nest, oropharyngeal trichomonosis by *Trichomonas gallinae* is the most important, which accounts up to 22% of mortality causes depending on the year [4], and in some years, up to 87.5% of the broods showed oropharyngeal lesions compatible with avian trichomonosis [5]. Oropharyngeal trichomonosis is one of the main causes of morbidity of wild birds in Spain [6]. More than 41% of the nests were infected with the parasite in some studies [4], while other studies displayed higher prevalence values, up to 54.5% of the eagles [7], and up to 45.5% of the analysed nestlings [8]. A situation that favours the transmission of oropharyngeal trichomonosis to the nestlings is attributed to columbiforms, the main reservoir of *T. gallinae*, since they constitute the preferred Bonelli’s eagle prey at present, displacing rabbits, and red-legged partridges [9].

It is well known that the microbiome and the complex interactions host-parasites-bacteria may be multidirectional and greatly influence the health status of the animals. Changes in the diet may alter the oral microbiome, which can be also influenced by host morphology and phylogeny, captivity, antibiotic treatment, age, sex, and the presence of certain pathogens, such as *T. gallinae* [10–13]. The interaction of parasites and bacteria has been also studied. For example, the presence of *Clostridium perfringens* during an infection by *Eimeria meleagrimitis* in turkeys worsens the pathological scenario [14], while it has also been suggested that the presence of *Eimeria* may alter the microbiota composition [15]. Whatever the mechanism, it seems clear that interactions between bacteria and parasites are highly probable.

The composition of the microbiome can also influence the outcome of the immune response [16, 17], including granuloma development during helminth infections [18]. Microbiome composition can also mediate colonisation-resistance against pathogens [19, 20].

While many studies on the microbiome have been carried out in mammals and humans, only a tenth have been done on the avian microbiome [21]. Even so, the microbiome of domestic birds, such as chickens or turkeys has been widely explored, but there is a scarce number of publications on wild bird species so far, and they are mainly focused in necrophagous birds [20, 22, 23]. However, some information has been published on other raptors, like the oral microbiome of Cooper’s hawk (*Accipiter cooperii*) [12], the intestinal microbiome of oriental honey buzzard (*Pernis ptilorhynchus*) [24] or the influence of sex and movement behaviour on barn owls’ (*Tyto alba*) microbiomes [25]. It is not well established yet if avian microbiomes from the same species or from closely related species could be as connected as in mammals, where the influence of the diet on the microbiome composition seems to be stronger than the genetic relatedness between different mammalian species [10]. For example, hominids with a plant-based diet display a microbiome which is intermediate between that of omnivorous hominids and that of Artiodactyls [10]. In this sense, microbiomes of the facial skin of scavenger birds are more similar to other scavengers’ facial microbiomes than to other bird facial microbiomes, including non-scavengers’ raptors [22].

In this context, several conservation actions, such as captivity breeding and sampling of chicks in nests to monitor oropharyngeal trichomonosis were carried out under the European project EU-LIFE12 NAT/ES/000701, actions that we complemented with a study of the oral microbiome employing the same samples of trichomonosis analysis.

This work describes for the first time the composition of the oral microbiome of Bonelli’s eagle chicks raised in the wild and the influence of factors, such as captivity breeding, *T. gallinae* infections or the presence of lesions in the oral cavity.

## Methods

### Samples

**Fig. 1:**
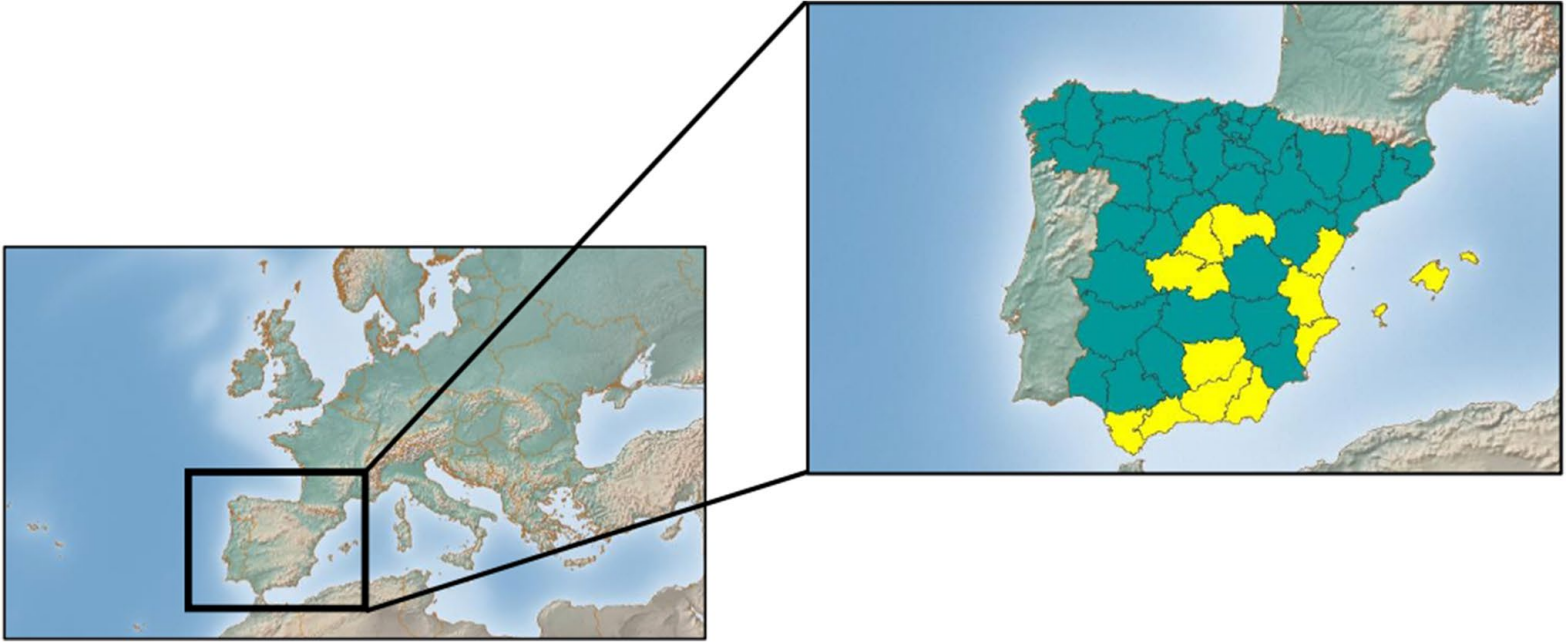
Origin of the samples obtained from Bonelli’s eagle chicks at nest in Spain (province areas in yellow)

Samples from 83 chicks were analysed. Twenty-seven of the animals were bred in captivity and the other 56 chicks were sampled at nests, when they were between 30 and 47 days of age, during the months of April and May. The nests were located in different geographic areas of Spain (Fig. 1), the most important breeding area of the Bonelli’s eagle European population. In total, 38 nests were sampled, representing 10.67% of nests with chicks of Spain [26], each one containing 1 or 2 chicks. Most of the nests were in places at high altitudes, and Mediterranean climate predominates, with hot and dry summers and cold winters. The selection of the nests was carried out depending on different criteria, such as accessibility, different nests each year and the extension of the provinces where the nests were located. In total, and considering only nests with chicks, the provinces sampled covered 66.3% of the breeding pairs of the country and the percentage of the nests sampled in each province varies from 11% of nests (in large nestling areas like Andalucía, Valencian Community or Castilla La Mancha) to 100% of nests (in smaller nesting areas like Madrid and Mallorca).

Oropharyngeal sterile swabs were aseptically taken from each chick and then kept frozen at − 20 °C until DNA extraction. A visual inspection of the oropharyngeal cavity was carried out to detect the presence of macroscopic lesions, and when found, they were classified as mild, moderate or severe, according to criteria previously published [27], such as the size of the lesion in relation to the tracheal opening, the depth (superficial or deep) and the location of the lesions (distance from the tracheal opening).

### DNA Isolation

Each swab was used for DNA extraction using the DNeasy Blood and Tissue Extraction Kit (QIAGEN, Valencia, CA, USA) [28]. Extracted DNA was eluted in 22 μL of nuclease-free water and stored at − 20 °C until further analysis. Purity and concentration of each extracted DNA sample were estimated using a NanoDrop 1000 spectrophotometer (NanoDrop Technologies, Inc., Rockland, USA). Negative controls (blanks) were also performed using nuclease-free molecular grade water (Sigma-Aldrich, St. Louis, MO) in the DNA extraction and purification process.

### Identification of *T. gallinae* by ITS Amplification and Sequencing

The employed oligonucleotide primers for the specific PCR amplification of the ITS region of *T. gallinae* were as follows: TFR1 (5′-TGCTTCAGTTCAGCGGGTCTTCC-3′) and TFR2 (5′-CGGTAGGTGAACCTGCCGTTGG-3′) [29]. PCR was done as previously described [29] in a GeneAmp 2700 thermal cycler (Applied Biosystems, Foster City, California, USA). Amplified products were analysed by electrophoresis in 1% agarose gels stained with SYBR Green and visualised under UV light in a transilluminator. Positive (*T. gallinae* genomic DNA) and negative (sterile water) controls were included in each PCR set.

Amplicons were sequenced by Sistemas Genómicos, S. A. (Paterna, Valencia, Spain) as described elsewhere [8] and sequences were compared with others from GenBank database (http://www.ncbi.nlm.nih.gov/genbank/).

### Metataxonomic Analysis

A fragment of the V3-V4 hypervariable region of the bacterial 16S ribosomal RNA gene was amplified by a dual-barcoded 2-step PCR. Equimolar concentrations of the universal primers S-D-Bact-0341-b-S-17 (ACACTGACGACATGGTTCTACACCTACGGGNGGCWGCAG) and S-D-Bact-0785-a-A-21 (TACGGTAGCAGAGACTTGGTCTGACTACHVGGGTATCTAATCC) were employed [30]. Barcodes used for Illumina sequencing were attached to 3′ and 5′ ends of the amplicons to allow the separation of forward and reverse sequences. PCR products were pooled at equimolar DNA concentrations and run on a preparative agarose gel. The bands were excised and purified using a QIAEX II Gel Extraction Kit (QIAGEN) and then, quantified with PicoGreen (BMG Labtech, Jena, Germany). Aliquots of the purified barcoded DNA amplicons were sequenced using the Illumina MiSeq pair-end protocol (Illumina Inc., San Diego, CA) at the facilities of Parque Científico de Madrid (Spain).

The data that support the findings of this study will be openly available in Metagenomic Resources (https://www.ncbi.nlm.nih.gov/genbank/metagenome/, PRJNA759868).

The sequences were demultiplexed using the Illumina software (version 2.6.2.3) according to the manufacturer’s guidelines and pipelines. Further bioinformatics analyses were performed combining QIIME 2 2019.1 [31] and the R software (version 3.5.1, https://www.r-project.org/) [32].

DADA2 pipeline [33] was used for denoising. The forward reads were truncated at position 285 and their first 12 nucleotides were trimmed, while the reverse ones were truncated at the position 240 and their first nine nucleotides were trimmed, to discard positions for which nucleotide median quality was Q20 or below.

Taxonomy data was assigned to amplicon sequence variants (ASVs) using the q2-feature-classifier [34] classify-sklearn naïve Bayes taxonomy classifier against the SILVA 138 reference database [35].

The decontam package version 1.2.1 [36] was used to identify, visualise and remove contaminating DNA with a negative control sample.

### Statistic and Bioinformatics Analysis

The Shannon diversity index [37] was performed with the R vegan package (version: 2.5.6) [38] and was employed to estimate alpha diversity, which considers the number and evenness of microbial species, with the Wilcoxon rank test to find statistical differences between groups, and values were expressed as median and quartiles 1–3 range (Q1–Q3). Beta diversity was studied using principal coordinates analysis (PCoA) to visually display patterns of beta diversity through a distance matrix containing a dissimilarity value for each pairwise sample comparison. For quantitative and qualitative analyses, the Bray–Curtis and the binary Jaccard indexes were used, respectively. Permutational multivariate ANOVA (PERMANOVA) with 999 permutations was employed. Heat maps and cladograms were performed with the Hclust hierarchical cluster analysis with complete linkage method from the R’s core package “stats” and the “ggplot2” package [39].

Correlation networks considering the 20 more abundant genera were performed using the Lasso method with a tuning of 0.25 employing the QGRAPH R package [40] and the Spearman’s rank correlation test was carried out to analyse relationships between genera. Differences in phyla and genera between groups of samples were analysed by comparison using the Wilcoxon rank test. Values of relative abundance were expressed as median, and quartiles 1–3 range (Q1–Q3) and statistical significance was considered with *p* value < 0.05.

Variables for the statistical analysis were type of breeding (captivity/nest), oropharyngeal lesions (presence/absence), severity of the lesions (mild/moderate/severe) and *T. gallinae* infection (presence/absence).

A high prevalence (80% of the samples) and a minimum relative abundance (0.01% in each sample) were set as requisites to consider a taxa as a member of the core microbiome in the chicks.

## Results

### Rates of *T. gallinae* Infection

*T. gallinae* was present in 37 of the samples, seven from captivity breeding chicks (*n* = 7/27, 25.9% prevalence) and the rest from chicks sampled at nest (*n* = 30/56, 53.6% prevalence by chicks, 21/38 nests infected, 55.3% prevalence by nests). Sequencing of the ITS region revealed 100% identity with EU881911 and EU881912, the two most common genotypes of *T. gallinae* in Bonelli’s eagle [7].

Mild oropharyngeal lesions were present in only one of the animals bred in captivity (*n* = 1/27, 3.7%). On the other hand, 18 animals at nests showed oropharyngeal lesions compatible with trichomonosis (*n* = 18/56, 32.1%), 15 mild (15/56, 26.8%) and three severe (*n* = 3/56, 5.4%).

### Metataxonomic Analysis

The analysis of the oropharyngeal swabs (*n* = 83) rendered 4,107,022 high quality reads corresponding to 2081 different ASVs. Overall, 22 phyla and 221 genera were identified. The dominant phyla were Firmicutes (the most abundant one), Proteobacteria, Bacteroidota, Fusobacteriota and Actinobacteriota.

#### Influence of Captivity Breeding on the Oral Microbiome

**Fig. 2:**
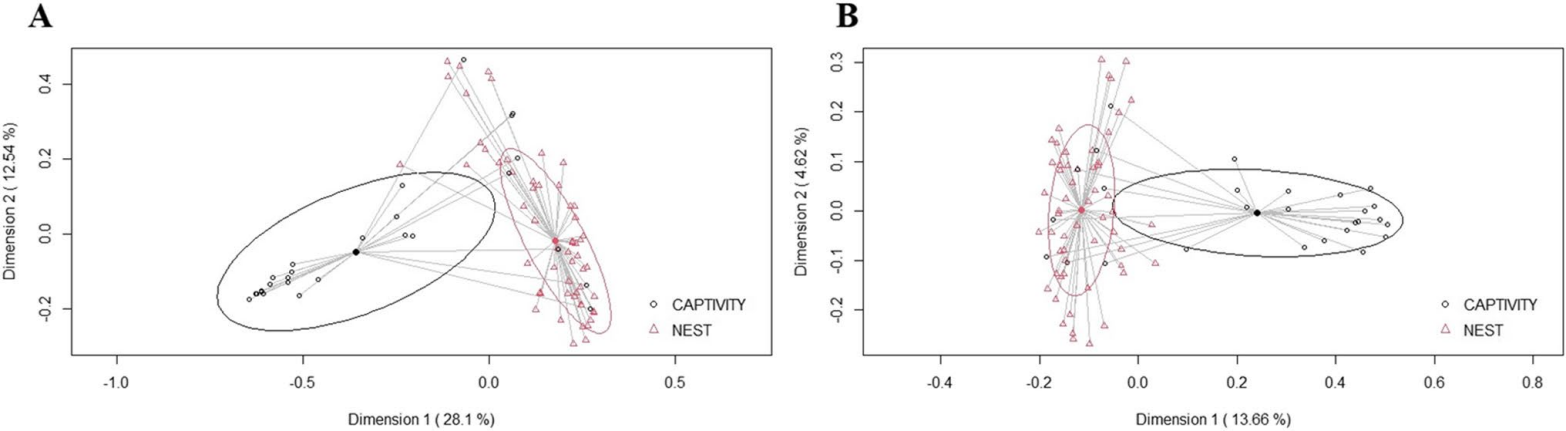
**A** Multiple PCoA quantitative analysis (relative abundance) displaying differences in the bacterial profile based on oropharyngeal swabs (*n* = 83) from chicks bred at nest (red, *n* = 56) and chicks bred in captivity (black, *n* = 27) (Bray-Courtis dispersion study). **B** Multiple PCoA qualitative analysis (*n* = 83) displaying differences in the presence/absence (Binary-Jaccard dispersion study) of bacteria from chicks bred at nest and chicks bred in captivity

**Fig. 3:**
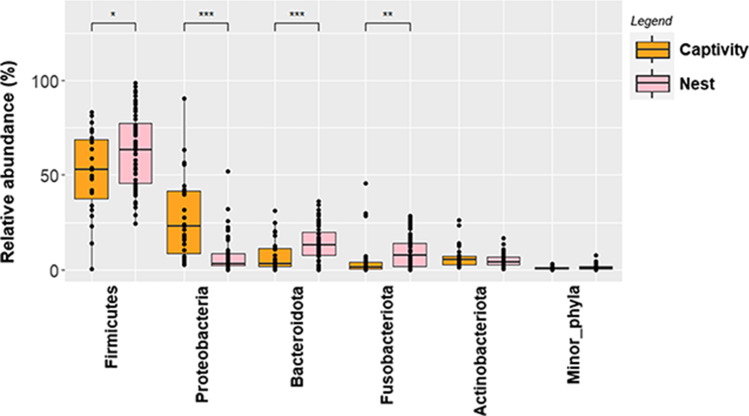
Changes in the relative abundance of the main bacterial phyla found in this study. Comparison between chicks bred at nest (pink) and chicks bred in captivity (orange) is shown. Wilcoxon rank tests with Bonferroni correction showed significant differences marked with asterisk(*: *p* < 0.05, **p < 0.01, ***p < 0.001)

Alfa diversity in the group of animals bred at nest (Shannon index = 2.92 [2.67–3.31]) was like that found in the group reared in captivity (Shannon index = 2.98 [2.80–3.31]). In contrast, the rearing method exerted a strong impact on beta diversity, both in terms of relative abundance (Bray-Courtis, *p* < 0.001) and presence/absence (Binary-Jaccard *p* < 0.001) (Fig. 2). Relevant differences in the composition of ASVs were also found between the group of animals bred in captivity and the group bred at nest. Firmicutes was the most abundant phylum in both groups, although it was more abundant in the nest-bred group (*p* = 0.037) (Fig. 3). On the contrary, the relative abundance of Proteobacteria was higher in the captivity-bred group (*p* < 0.001). The abundance of Bacteroidota and Fusobacteria also differed between both groups, being higher in the group of chicks bred at nest (*p* < 0.001 and *p* = 0.002, respectively). No differences were observed between both groups regarding the relative abundance of the phylum Actinobacteriota (*p* = 0.4). Campylobacteriota, one of the minor phyla, was detected in approximately 80% of the samples, but at a very low relative abundance (0.1%), while this phylum was absent in the core of the samples from the captivity-bred group.

**Fig. 4:**
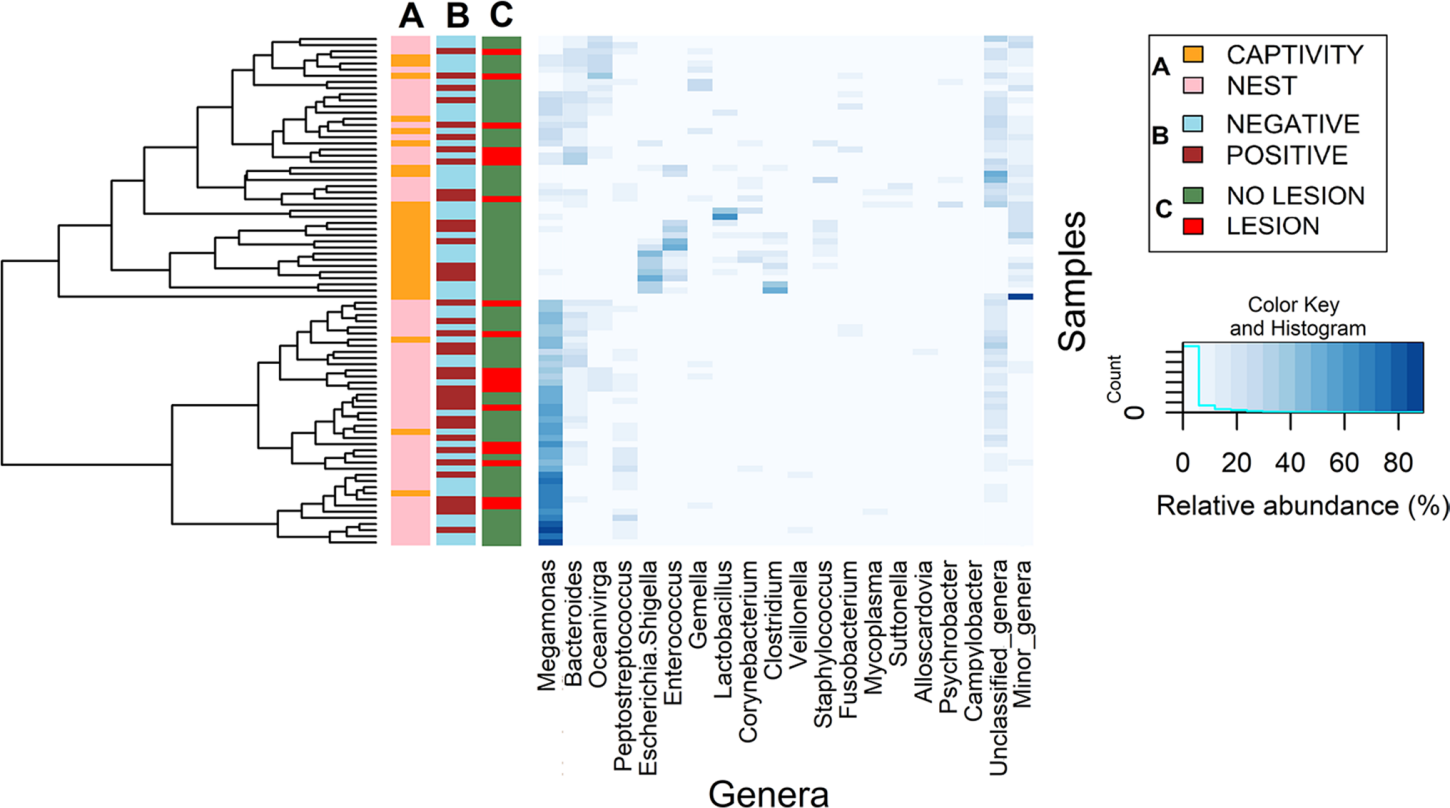
Heatmap showing the difference in genera abundance considering the main factors of influence analysed: **A** type of breeding (captivity/nest), **B** *T. gallinae* infection (positive/negative) and **C** oropharyngeal lesions (lesion/no lesion). The cladograms were performed with the Hclust hierarchical cluster analysis with complete linkage method. The intensity of the colour reflects the abundance of each genus

At the genus level, significant differences were also observed between both groups (Supplementary Table 1; Fig. 4). *Megamonas* was the most abundant genus but was more abundant in the nest-bred group, where it represented 40.62% (23.24–54.51) of the identified bacterial sequences, than in the captivity-bred group, where it accounted for 2.04% (0.92–13.92) of the sequences. Other genera more abundant in the nest-bred group included *Bacteroides*, *Oceanivirga*, *Peptostreptococcus*, *Gemella*, *Veillonella*, *Mycoplasma*, *Suttonella*, *Alloscardovia*, *Varibaculum* and *Campylobacter.* On the contrary, *Escherichia*-*Shigella*, *Enterococcus*, *Lactobacillus*, *Corynebacterium*, *Clostridium* and *Staphylococcus* were more abundant in the group of captivity-bred animals (Supplementary Table 1; Fig. 4). The only genus with a similar proportion in both groups of chicks was *Fusobacterium*.

**Fig. 5:**
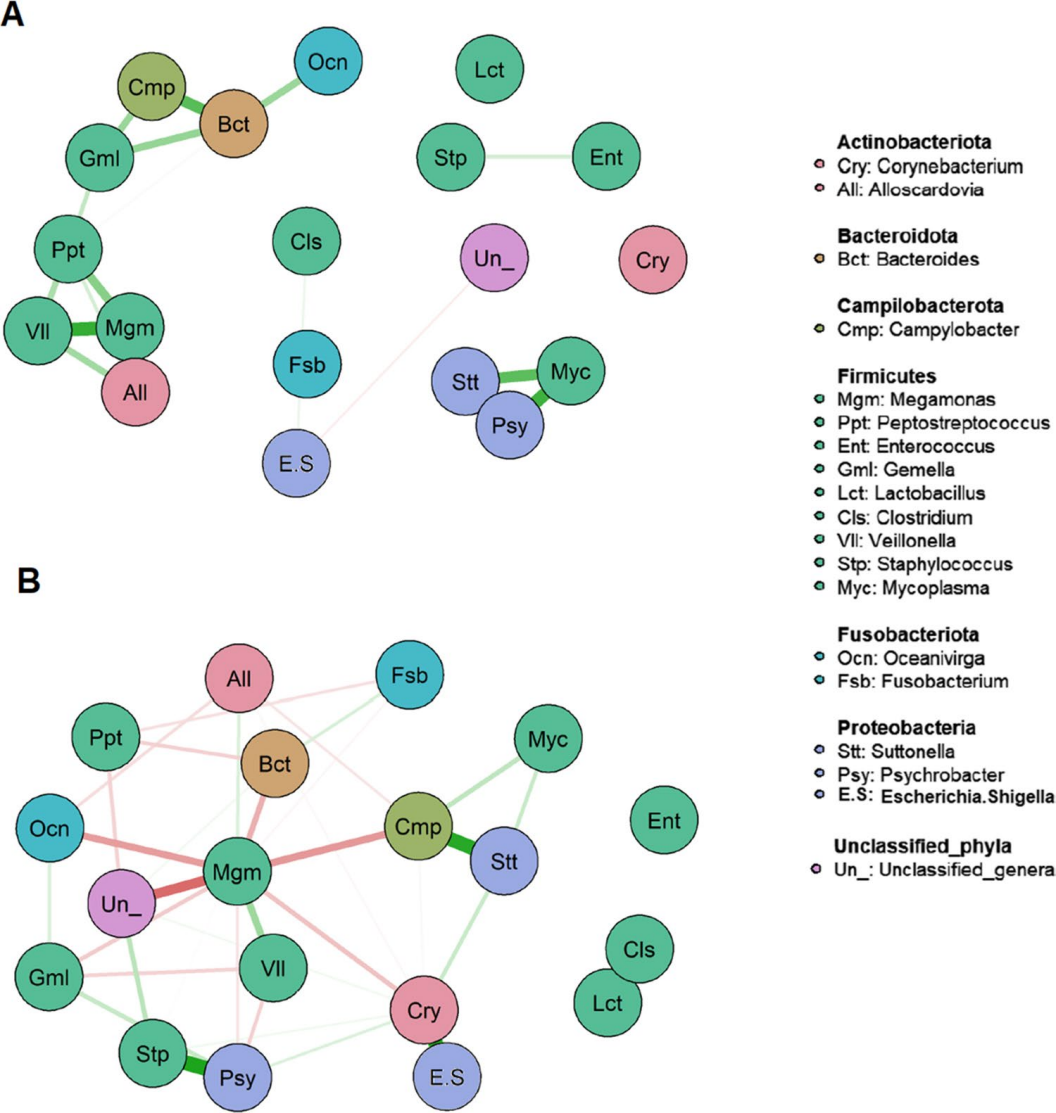
Network indicating the correlation among bacteria genera from chicks in nest (**A**) and the correlation among bacteria genera in chicks raised in captivity (**B**). Green edges mean positive correlation while red edges mean negative correlation. The edge size senses the strength of the correlation

Networking analysis of the 20 more abundant genera showed that association among them was far more complex in samples from birds raised at nest (which was very diverse in origin) than in samples from captivity-raised birds (which came from the same place and received a similar diet) (Fig. 5). Captivity-raised birds showed a strong and positive correlation between a smaller number of genera (thick green lines), while birds raised at nest displayed many connections among different genera, both positive (green colour) and negative (red colour), although connecting lines were thinner, which can be interpreted as a higher diversity and variability among chicks.

#### Influence of *Trichomonas* Infection on the Oral Microbiome

Subsequently, the influence of *T. gallinae* infection on the diversity and composition of the bacteriome in chicks bred at nest was assessed (Supplementary Table 2). No differences in alfa or beta diversity were observed between the groups of infected and uninfected animals.

In relation to the influence of *T. gallinae* infection in nestlings bred in captivity, alfa diversity did not differ between infected and uninfected chicks (Shannon index values of 2.96 [2.68–3.23] and 3.0 [2.84–3.3], respectively). The analysis of beta diversity did not influence the relative abundance nor the presence/absence of the most abundant phyla (Supplementary Fig. S1), but differences in the relative abundance of two minor phyla were observed. The phylum Planctomycetota was present in the core of the *T. gallinae*-infected chicks bred in captivity but not in the uninfected, and the phylum Campylobacteriota appeared in the uninfected birds in contrast to *T. gallinae*-infected chicks (Supplementary Fig. S2A).

*Trichomonas*-infected chicks bred in captivity had higher abundances of *Staphylococcus* and *Enterococcus* than uninfected chicks (*p* = 0.033 and *p* = 0.015, respectively); a similar trend was observed for *Escherichia-Shigella* although it did not reach a statistically significant value (Supplementary Table S3).

*Campylobacter* was the only genus present in the core of uninfected chicks in comparison with *T. gallinae*-infected birds. Oppositely, many genera were found in 80% of the *Trichomonas*-infected group among captivity-bred chicks, in comparison with uninfected birds; *Clostridium*, *Oceanivirga*, *Gemella*, *Fusobacterium*, *Ralstonia*, *Lactococcus* and *Alloscardovia* (0.01% relative abundance); *Peptostreptococcus*, *Proteus, Streptococcus* and *Kocuria* (0.1% relative abundance) and *Escherichia-Shigella*, *Enterococcus*, *Lactobacillus*, *Staphylococcus* and *Corynebacterium* (1% relative abundance) (Supplementary Fig. S2B).

#### Influence of Lesions on the Oral Microbiome

Finally, the influence of the presence of lesions on the oropharyngeal bacteriome of the chicks bred at nest was evaluated. No differences in alfa diversity were observed between the groups of animals bred at nest with or without oropharyngeal lesions (Shannon index values of 3.02 [2.71–3.31] and 2.9 [2.64–3.3], respectively). In relation to beta diversity, no differences between both groups of chicks were detected regarding the relative abundance (*p* = 0.168) but significant differences were observed in terms of presence/absence (*p* = 0.048, Supplementary Fig. S3; Supplementary Table S4).

There were no differences in the abundance of the main detected phyla between both groups although the abundance of the phylum Bacteroidetes tended to be higher in the group of birds displaying lesions (Supplementary Fig. S4).

At the genus level, the abundance of *Bacteroides* and *Gemella* in the samples from chicks with lesions was higher than in those from chicks without oropharyngeal lesions (*p* = 0.037 and *p* = 0.011, respectively) (Supplementary Table S4). Besides, the genus *Oceanivirga* was present in the core of birds with lesions, with 1% relative abundance, in contrast to the observations made in birds without lesions.

In this study, only three animals displayed severe lesions of trichomonosis, according to the above-mentioned criteria [27]. The analyses of alfa and beta diversity did not show differences between birds severely affected by the parasite and those with mild or no oropharyngeal lesions, although a trend towards a lower beta diversity was observed in animals with severe lesions (*p* = 0.079). The phylum Fusobacteriota was detected in most of the animals with severe lesions (≥ 80%) in contrast to the group of animals with mild or no lesions. The abundance of *Gemella* and *Ornithobacterium* in the core of birds with severe lesions was higher than in the rest of the birds bred at nest (*p* = 0.026 and *p* = 0.026, respectively). The relative abundance of *Gemella* was 1.12 (0.43–3.11) in birds with mild or no lesions compared to 7.15 (5.44–8.97) in birds severely affected by trichomonosis, while *Ornithobacterium* appeared with a relative abundance of 0.21 (0.06–0.51) in birds with mild or no lesions vs. 1.49 (0.98–3.03) in birds severely affected by trichomonosis.

## Discussion

The overall composition of the oral bacteriome of Bonelli’s eagles bred at nest is similar to that reported for Cooper’s hawk [12]. Four of the most abundant phyla, Firmicutes, Bacteroidota, Proteobacteria and Actinobacteria are also common in the oral microbiome of other vertebrates, such as dogs, cats and humans [12]. In addition, Firmicutes and Bacteroidota, the most abundant phyla in the oropharynx of Bonelli’s eagles’ chicks in nature, are frequently detected in the gut of reptiles, birds, and mammals, which share common ancestors [41]. In fact, Bacteroidetes, Proteobacteria and Firmicutes conform the core of the vertebrate microbiome, as suggested by some authors, although captivity may have an impact on their frequency of detection and relative abundance [42].

Fusobacteriota was the third most abundant phylum in chicks from nests in our study. They are anaerobic microbes that metabolise amino acids better than sugars, frequent in the anterior digestive tract of carnivorous species, and they conform up to 5% of the oral human microbiome [42]. Although it was present also in the oral cavity of Cooper’s hawk, its relative abundance was much lower [12]. When the digestive microbiome of several species of birds was studied, a clear influence of the diet was detected since the numbers of Fusobacteria and Proteobacteria sequences were higher in carnivorous birds in comparison with birds on plant fibre- or starch-based diets, which, in turn, showed higher amounts of *Clostridium* and *Lactobacillus* reads, respectively [43]. Bonelli’s eagle is a carnivorous species, and this fact may explain the relative abundance of Fusobacteria in the samples analysed in this work.

A similar composition was observed also in the gut microbiome of several avian species, in which the four phyla mentioned above predominated [43]. In the gut of common kestrel (*Falco tinnunculus*), Proteobacteria, Firmicutes, Actinobacteria and Bacteroidetes also predominate [44]. Other authors found similar results, with the same four phyla displaying high abundancies, and no significant differences in the diversity of the faecal microbiome of Strigiformes, Accipitriformes and Falconiformes [45]. In this last study, the authors also described values of Fusobacteria abundance higher than 1%.

The genera *Megamonas* and *Bacteroides* were predominant within the bacteriome of chicks bred at nest. Both are common inhabitants of the gut of several avian species and some factors, including the gender or diet of the animals, may have an impact on them [46, 47]. In fact, the presence of *Megamonas* and *Bacteroides* was highly affected by captivity breeding in this study. Other genera described in Bonelli’s eagle chicks, such as *Sutonella* and *Veillonella*, are also present in the oral cavity of Cooper’s hawk [12].

According to some authors, microbiome studies of captive vertebrates, including birds, should not be extrapolated to wild populations [48]. Other studies support the idea that the phylogenetic influence may be stronger than captivity-related changes (diet, environment) in shaping the bacteriome [42]. In this context, our results support the first hypothesis since the type of breeding was the most relevant factor affecting the oropharyngeal bacteriome of Bonelli’s chicks. We found more diversity in the microbiome of chicks bred at nest than in chicks bred in captivity, which have a more homogeneous diet. Differences can also be due to the components of the nest that are absent in captivity bred chicks; as an example, kestrels have higher abundance of Proteobacteria than Cooper’s hawks, probably due to worse hygienic conditions [39]. Besides, feathers or plants present in nest may have antibacterial properties [49].

At genus level in our study, *Campylobacter* was one of the genera more abundant in chicks bred at nest than chicks under captivity breeding. This genus is a commensal in chicks, and it is more frequent in the avian gut than in the human gut, probably due to the difference in mucin composition [41]. Probably the human influence on the oral microbiome of the captivity-raised chicks had consequently decreased some genera that are common in birds, such as *Campylobacter*.

A time-dependent increase in Actinobacteria in the bacteriome of birds kept in captivity was previously found [45]. In our study, Firmicutes, Bacteroidota and Fusobacteria were more abundant in birds bred at nest while the contrary happened with Proteobateriota. However, Actinobacteriota did not differ between both groups. Differences between studies might be due to several reasons, including the avian species, time of the year, anatomical location (oropharyngeal vs. faecal samples) or potential human contamination during avian feeding or sampling [50].

Among birds bred at nest, metataxonomic differences between animals infected or uninfected with *T. gallinae* were small. In contrast, some differences were found when the impact of the infection was assessed in birds bred in captivity. More specifically, Planctomycetota was found in a higher percentage of chicks infected with the parasite. This phylum is frequently found in different types of water, including marine, freshwater and wastewater treatment plants, among others [51], and the transmission of *T. gallinae* is favoured by sharing water, a fact that can explain its association with the parasite. Since all our samples were subjected to DNA isolation simultaneously, we can exclude an external contamination after sampling, and reinforce the hypothesis of an association between water contamination and *T. gallinae* infection.

A significant effect of *T. gallinae* infection on the richness of the microbiome in the crop of infected pigeons at 14 days of age was previously found [13]. The influence of the parasite was evident in the abundance of several genera in the small intestine at 21 days of age, with a lower relative abundance of *Lactobacillus* and higher of *Enterococcus* and other genera in *T. gallinae*-infected pigeons. In our study, an increase not only in *Enterococcus* but, also, in *Lactobacillus* was found in *T. gallinae*-infected birds. This may reflect the influence of human handling of the avian food since animals were kept in captivity in both cases. However, further comparisons between both studies are difficult since they implied different avian species, feeding habits and type of breeding. Also, *T. gallinae* infected pigeons showed lower alfa diversity in the gut and rectum [13]. In our study, a tendency to decrease alfa diversity was observed, but with no significant difference, probably due to the low number of animals analysed.

Chicks bred at nests were more frequently infected by *T. gallinae* and displayed moderate or severe lesions at the oropharynx. Since the diet of the chicks bred in captivity was more homogeneous, the exclusion of *T. gallinae* under this condition is more probable. Besides, the transmission of the parasite is highly associated with the increase of columbids in the diet [9], which are not included in captivity bred chicks’ diet.

Four genera (*Bacteroides*, *Gemella*, *Oceanivirga* and *Ornithobacteria*) were somehow associated with the development of *T. gallinae*-related lesions. *Bacteroides*, *Gemella* and *Oceanivirga* were found in higher relative amounts in animals with mild or severe lesions.

When severely affected animals (*n* = 3) were compared to the rest of animals, a significant increase was observed again in the relative amount of *Gemella*. This genus inhabits the oral mucosal surface and is an opportunistic pathogen associated with the development of inflammation and abscesses in several locations [52, 53]. It is remarkable that *Gemella* was found seven times more abundant in birds developing severe oropharyngeal lesions. When these lesions tend to coalesce and aggravate, abscesses can be seen in severely affected birds, similarly to what has been described in human cases of endocarditis, meningitis and orbital or maxillary abscesses [52, 53].

Although many factors may potentially exert an influence on the composition of the oral bacteriome, a study focused on the Cooper’s hawk’s microbiome did not find differences according to the date of sampling or the location of the hawks [12]. Some authors found differences in the digestive tract microbiome related to age in pigeons and kestrels [13, 44]. Studies dealing with the factors affecting the raptors’ microbiome are very scarce, but diet is, most probably, the main factor driving the composition of the avian microbiome, since its influence seems to be much stronger than that of host phylogeny, weather, season, sex, age or geographic location [43].

In conclusion, the result of this study shows that captivity leads to structural changes in the oropharyngeal bacteriome of Bonelli’s eagle and that the abundance of *Gemella* may be associated with the presence of mild or severe oropharyngeal lesions when *T. gallinae* infections occurs. Future studies on Accipitriformes comparing healthy birds with birds displaying oropharyngeal lesions due to *T. gallinae* infection are desirable to confirm our findings.

## Supplementary Information

Below is the link to the electronic supplementary material.

Supplementary file1 **Supplementary Fig. S1A **Multiple PCoA quantitative analysis (relative abundance) of oropharyngeal swabs (n=27) from chicks bred in captivity infected (red) or not (black) with *T. gallinae* (Bray-Courtis dispersion study, p<0.001). **Supplementary Fig. S1B** Multiple PCoA qualitative analysis showing the presence/absence in oropharyngeal swabs (n=27) from chicks bred in captivity infected (red) or not (black) with *T. gallinae* (Binary-Jaccard dispersion study) (PDF 20.1 KB)

Supplementary file2 ** Supplementary Fig. S2A**: Venn diagram representing the bacterial profile core based on shared membership using the phyla table of occurrences of the *T. gallinae*-infected (*T. gallinae* +) and non-infected (*T. gallinae* -) chicks bred in captivity from a total of 22 different phyla (number up to the right represent the total of phyla minus the phyla in the diagram). A high prevalence (80% of the samples) and a minimum relative abundance (A) (0.001% in each sample), (B) (0.01% in each sample) and (C) (0.1% in each sample) were set as requisites to consider a taxa as a member (YES) of the core microbiome in the chicks. The taxa that do not fulfil the criteria were not considered as a member of the core microbiome of the chicks (NO). **Supplementary Fig. S2B**: Venn diagram representing the bacterial profile core based on shared membership using the genera table of occurrences of the *T. gallinae*-infected (*T. gallinae* +) and non-infected (*T. gallinae* -) chicks bred in captivity from a total of 195 different genera (number up to the right represent the total of genera minus the genera in the diagram). A high prevalence (80% of the samples) and a minimum relative abundance (A) (0.001% in each sample), (B) (0.01% in each sample) and (C) (0.1% in each sample) were set as requisites to consider a taxa as a member (YES) of the core microbiome in the chicks. The taxa that do not fulfil the criteria were not considered as a member of the core microbiome of the chicks (NO) (PDF 291 KB)

Supplementary file3 ** Supplementary Fig. S3A **Multiple PCoA quantitative analysis (relative abundance) of oropharyngeal swabs (n=56) from chicks bred in nest with (red) or without (black) oropharyngeal lesions (Bray-Courtis dispersion study, p=0.168). **Supplementary Fig. S3B** Multiple PCoA qualitative presence/absence analysis of bacteria from oropharyngeal swabs (n=56) from chicks bred in nest with (red) or without (black) oropharyngeal lesions (Binary-Jaccard dispersion study, p=0.048) (PDF 20.8 KB)

Supplementary file4 ** Supplementary Fig. S4** Relative abundances of the main phyla found in chicks bred at nest with (yellow) or without (blue) oropharyngeal lesions. Wilcoxon rank tests with Bonferroni correction shown no statistical significance. (PDF 25.6 KB)

Supplementary file5 (PDF 102 KB)

Supplementary file6 (PDF 124 KB)

Supplementary file7 (PDF 101 KB)

Supplementary file8 (PDF 108 KB)

## Data Availability

The datasets generated during the current study are available in the SRA repository (https://www.ncbi.nlm.nih.gov), Bioproject Accesion Number PRJNA759868 (https://www.ncbi.nlm.nih.gov/bioproject/PRJNA759868).
