# Supplementary figures and images for: Captive Breeding and Trichomonas gallinae Alter the Oral Microbiome of Bonelli’s Eagle Chicks
Source: Microb Ecol. 2022 Apr 7;85(4):1541–51. doi: 10.1007/s00248-022-02002-y (PMC10167124; doi:10.1007/s00248-022-02002-y)

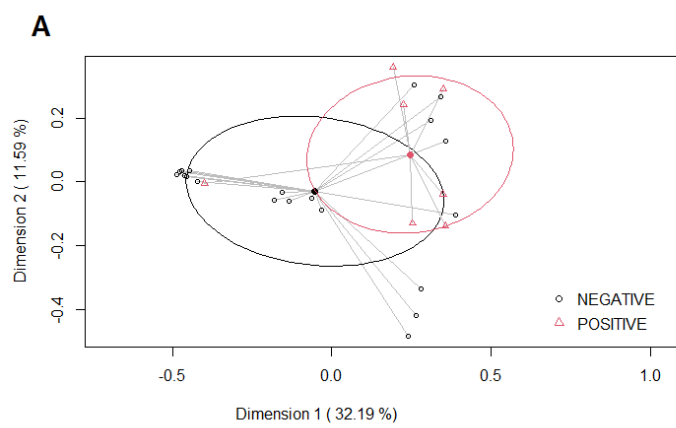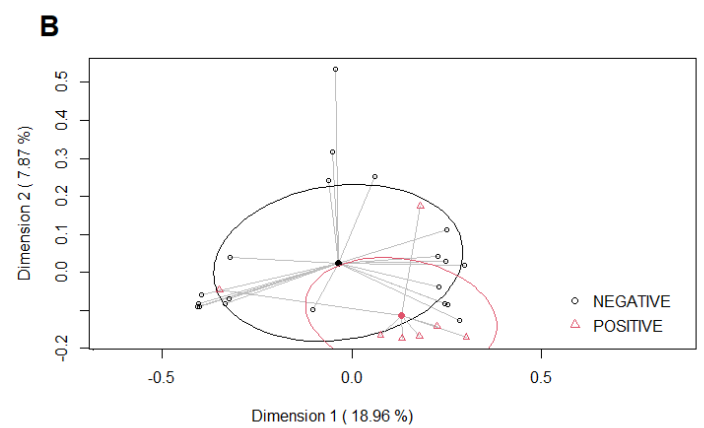

Supplement: Supplementary file 1 — Supplementary Fig. S1A Multiple PCoA quantitative analysis (relative abundance) of oropharyngeal swabs (n=27) from chicks bred in captivity infected (red) or not (black) with T. gallinae (Bray-Courtis dispersion study, p<0.001). Supplementary Fig. S1B Multiple PCoA qualitative analysis showing the presence/absence in oropharyngeal swabs (n=27) from chicks bred in captivity infected (red) or not (black) with T. gallinae (Binary-Jaccard dispersion study) (PDF 20.1 KB) [file 248_2022_2002_MOESM1_ESM.pdf]

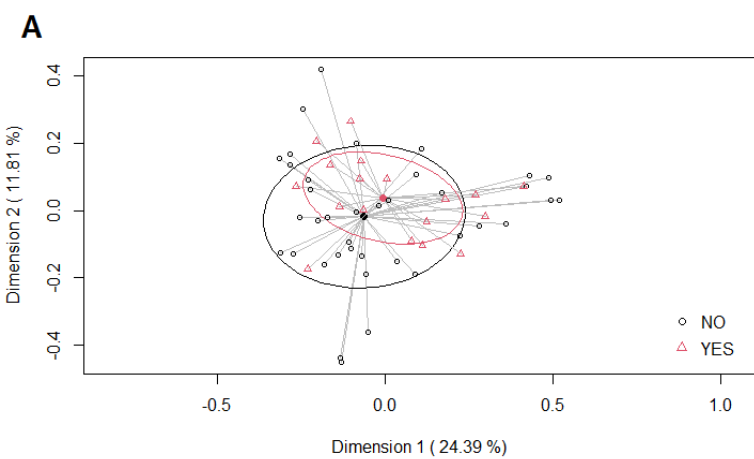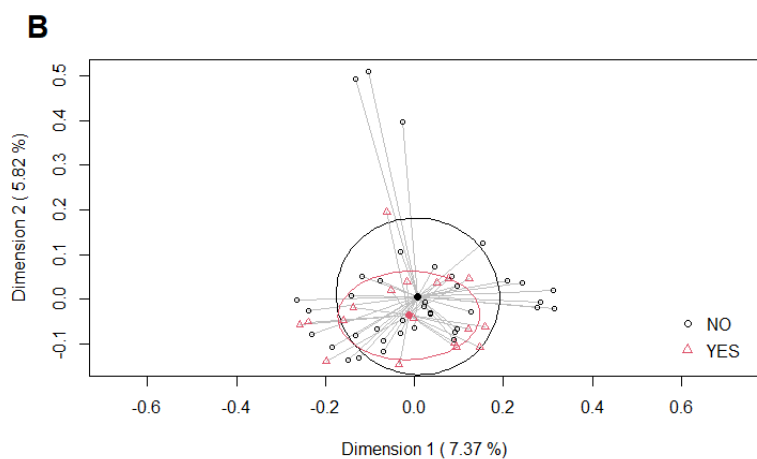

Supplement: Supplementary file 3 — Supplementary Fig. S3A Multiple PCoA quantitative analysis (relative abundance) of oropharyngeal swabs (n=56) from chicks bred in nest with (red) or without (black) oropharyngeal lesions (Bray-Courtis dispersion study, p=0.168). Supplementary Fig. S3B Multiple PCoA qualitative presence/absence analysis of bacteria from oropharyngeal swabs (n=56) from chicks bred in nest with (red) or without (black) oropharyngeal lesions (Binary-Jaccard dispersion study, p=0.048) (PDF 20.8 KB) [file 248_2022_2002_MOESM3_ESM.pdf]

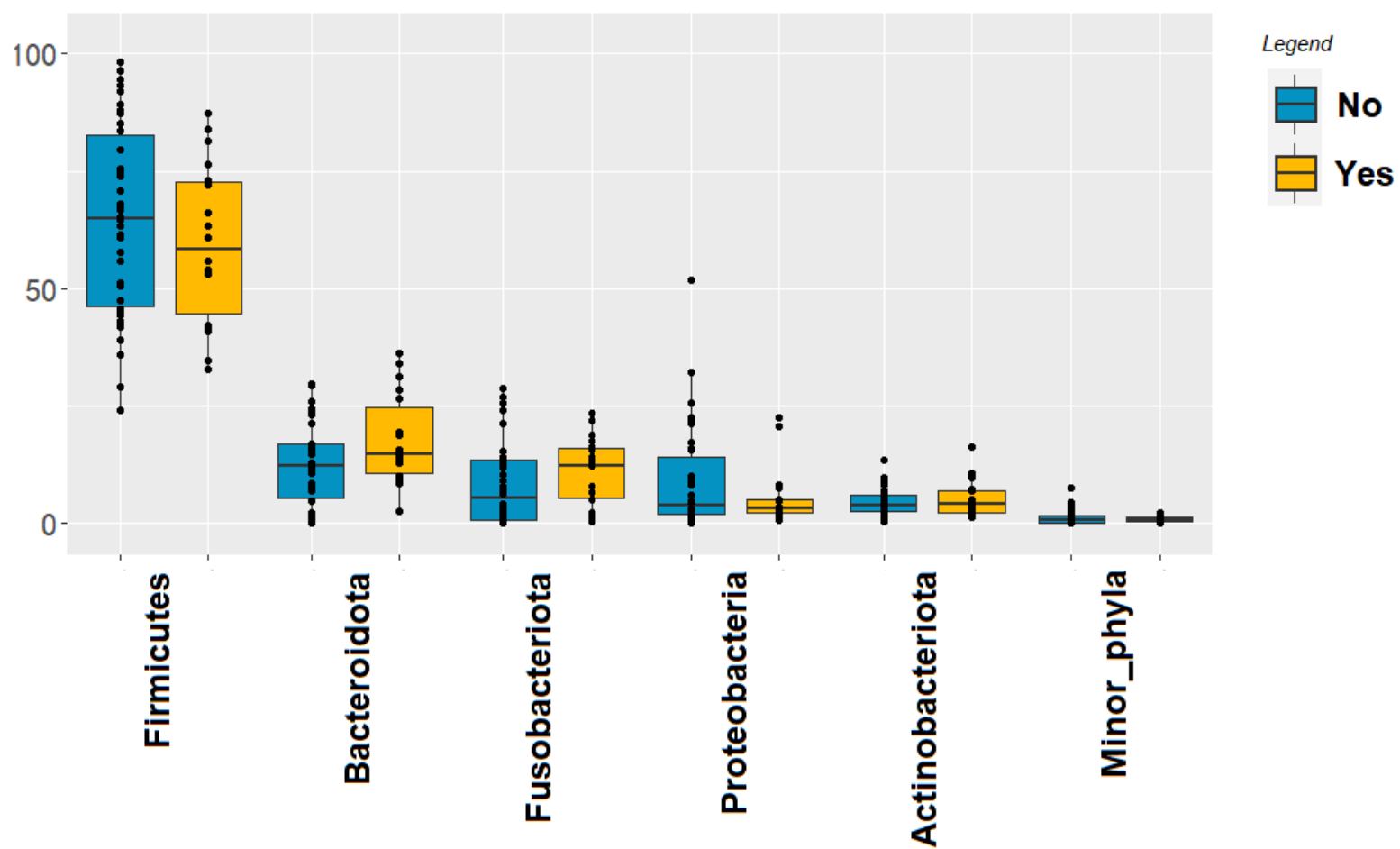

Supplement: Supplementary file 4 — Supplementary Fig. S4 Relative abundances of the main phyla found in chicks bred at nest with (yellow) or without (blue) oropharyngeal lesions. Wilcoxon rank tests with Bonferroni correction shown no statistical significance. (PDF 25.6 KB) [file 248_2022_2002_MOESM4_ESM.pdf]
