# Supplementary material for: Captive Breeding and Trichomonas gallinae Alter the Oral Microbiome of Bonelli’s Eagle Chicks
Source: Microb Ecol. 2022 Apr 7;85(4):1541–51. doi: 10.1007/s00248-022-02002-y (PMC10167124; doi:10.1007/s00248-022-02002-y)
Supplement: Supplementary file 2 — Supplementary Fig. S2A: Venn diagram representing the bacterial profile core based on shared membership using the phyla table of occurrences of the T. gallinae-infected (T. gallinae +) and non-infected (T. gallinae -) chicks bred in captivity from a total of 22 different phyla (number up to the right represent the total of phyla minus the phyla in the diagram). A high prevalence (80% of the samples) and a minimum relative abundance (A) (0.001% in each sample), (B) (0.01% in each sample) and (C) (0.1% in each sample) were set as requisites to consider a taxa as a member (YES) of the core microbiome in the chicks. The taxa that do not fulfil the criteria were not considered as a member of the core microbiome of the chicks (NO). Supplementary Fig. S2B: Venn diagram representing the bacterial profile core based on shared membership using the genera table of occurrences of the T. gallinae-infected (T. gallinae +) and non-infected (T. gallinae -) chicks bred in captivity from a total of 195 different genera (number up to the right represent the total of genera minus the genera in the diagram). A high prevalence (80% of the samples) and a minimum relative abundance (A) (0.001% in each sample), (B) (0.01% in each sample) and (C) (0.1% in each sample) were set as requisites to consider a taxa as a member (YES) of the core microbiome in the chicks. The taxa that do not fulfil the criteria were not considered as a member of the core microbiome of the chicks (NO) (PDF 291 KB) [file 248_2022_2002_MOESM2_ESM.pdf]

2A

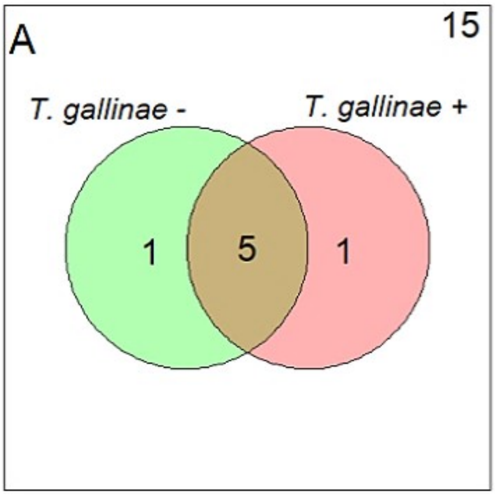

Phylum	*T. gallinae -*	*T. gallinae +*
Firmicutes	YES	YES
Proteobacteria	YES	YES
Bacteroidota	YES	YES
Actinobacteriota	YES	YES
Fusobacteriota	YES	YES
Campylobacteriota	YES	NO
Planctomycetota	NO	YES

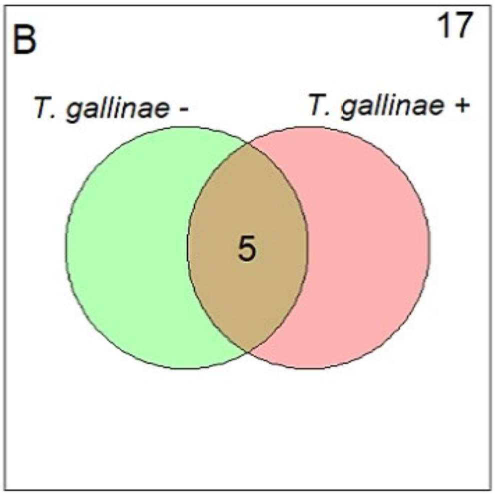

Phylum	*T. gallinae -*	*T. gallinae +*
Firmicutes	YES	YES
Proteobacteria	YES	YES
Bacteroidota	YES	YES
Actinobacteriota	YES	YES
Fusobacteriota	YES	YES

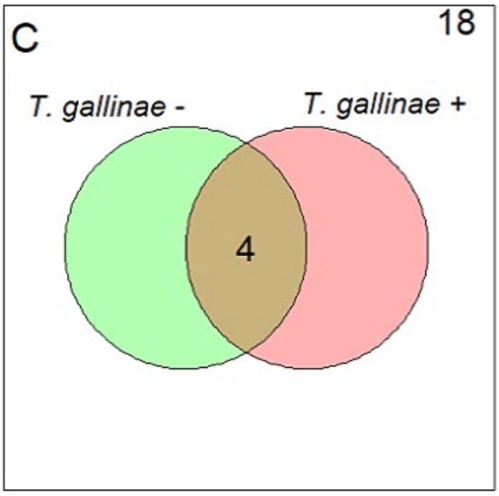

Phylum	*T. gallinae -*	*T. gallinae +*
Firmicutes	YES	YES
Proteobacteria	YES	YES
Bacteroidota	YES	YES
Actinobacteriota	YES	YES

2B

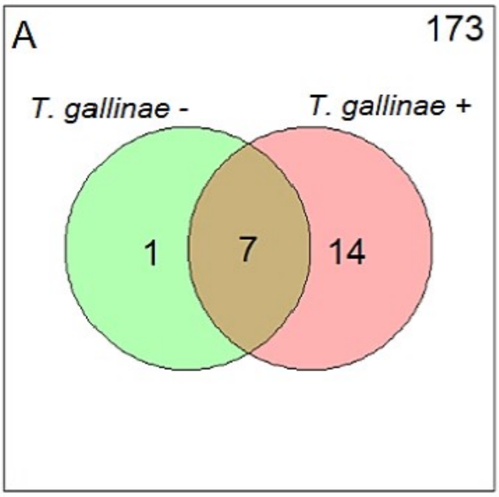

Genus	*T. gallinae -*	*T. gallinae +*
Escherichia-Shigella	NO	YES
Enterococcus	NO	YES
Megamonas	YES	YES
Unclassified_genera	YES	YES
Clostridium	NO	YES
Lactobacillus	YES	YES
Bacteroides	YES	YES
Staphylococcus	NO	YES
Corynebacterium	YES	YES
Oceanivirga	NO	YES
Peptostreptococcus	YES	YES
Gemella	NO	YES
Proteus	NO	YES
Streptococcus	NO	YES
Fusobacterium	NO	YES
Veillonella	YES	YES
Kocuria	NO	YES
Ralstonia	NO	YES
Campylobacter	YES	NO
Vibrionimonas	NO	YES
Lactococcus	NO	YES
Alloscardovia	NO	YES

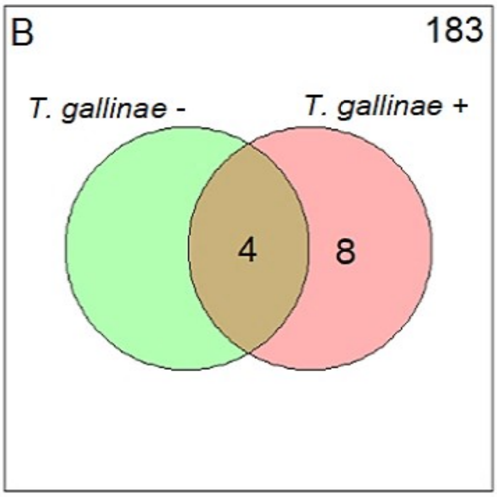

Genus	*T. gallinae -*	*T. gallinae +*
Escherichia-Shigella	NO	YES
Enterococcus	NO	YES
Megamonas	YES	YES
Unclassified_genera	YES	YES
Lactobacillus	NO	YES
Bacteroides	YES	YES
Staphylococcus	NO	YES
Corynebacterium	YES	YES
Peptostreptococcus	NO	YES
Proteus	NO	YES
Streptococcus	NO	YES
Kocuria	NO	YES

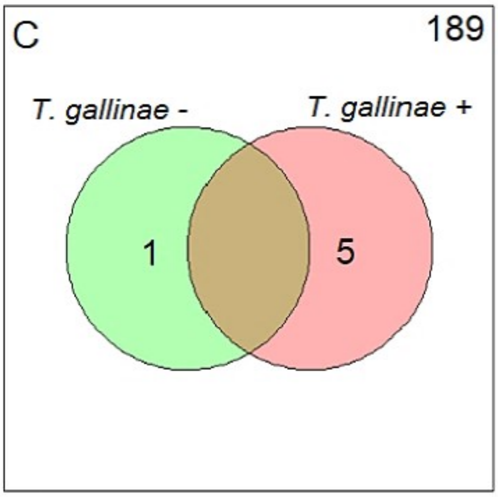

Genus	*T. gallinae -*	*T. gallinae +*
Escherichia-Shigella	NO	YES
Enterococcus	NO	YES
Unclassified_genera	YES	NO
Lactobacillus	NO	YES
Staphylococcus	NO	YES
Corynebacterium	NO	YES
