# Supplementary material for: Captive Breeding and Trichomonas gallinae Alter the Oral Microbiome of Bonelli’s Eagle Chicks
Source: Microb Ecol. 2022 Apr 7;85(4):1541–51. doi: 10.1007/s00248-022-02002-y (PMC10167124; doi:10.1007/s00248-022-02002-y)
Supplement: Supplementary file 5 — (PDF 102 KB) [file 248_2022_2002_MOESM5_ESM.pdf]

Supplementary Table S1. Relative frequencies, medians, and interquartile range (IQR) of the most abundant bacterial phyla (in bold) and genera detected in birds bred in captivity and chicks bred at nest ordered by relative abundance. **: Significant p values (lower than 0.05)

	Captivity-bred group		Nest-bred group		P-value†
**Phylum/Genus**	**n (%)[#]**	**Median (IQR)**	**n (%)[#]**	**Median (IQR)**	
**Firmicutes**	27 (100%)	52.96 (37.3-68.65)	56 (100%)	63.29 (45.57-75.66)	0.210
Megamonas **	26 (96%)	2.04 (0.42-13.92)	56 (100%)	39.19 (21.37-54.51)	<0.001
Peptostreptococcus **	24 (89%)	0.7 (0.11-3.81)	56 (100%)	5.16 (2.79-10.29)	<0.001
Enterococcus **	20 (74%)	2.68 (0.01-21.15)	11 (20%)	<0.01 (<0.01-<0.01)	<0.001
Gemella **	17 (63%)	0.1 (<0.01-2.48)	51 (91%)	1.26 (0.49-3.43)	0.004
Clostridium **	19 (70%)	0.51 (<0.01-7.55)	14 (25%)	<0.01 (<0.01-<0.01)	<0.001
Lactobacillus **	24 (89%)	1.62 (0.13-6.15)	37 (66%)	0.02 (<0.01-0.2)	<0.001
Veillonella **	25 (93%)	0.27 (0.07-1.23)	54 (96%)	2.36 (1.72-3.33)	<0.001
Staphylococcus **	23 (85%)	2.13 (0.05-7.08)	28 (50%)	<0.01 (<0.01-0.02)	<0.001
Mycoplasma **	15 (56%)	0.07 (<0.01-0.80)	55 (98%)	0.91 (0.45-1.93)	<0.001
**Proteobacteria ** **	27 (100%)	23.15 (8.68-41.65)	56 (100%)	3.23 (2.12-9.32)	<0.001
Escherichia,Shigella **	19 (70%)	2.52 (<0.01-26.63)	7 (12%)	<0.01 (<0.01-<0.01)	<0.001
Suttonella **	12 (44%)	<0.01 (<0.01-0.55)	54 (96%)	0.41 (0.19-1.14)	<0.001
**Bacteroidota ** **	26 (96%)	3.13 (1.55-11.05)	56 (100%)	12.95 (8.08-19.87)	<0.001
Bacteroides **	25 (93%)	1.67 (0.49-7.97)	56 (100%)	10.09 (6.45-14.65)	<0.001
**Fusobacteriota ** **	25 (93%)	1.38 (0.17-3.95)	56 (100%)	7.84 (1.61-14.49)	<0.001
Oceanivirga **	19 (70%)	0.31 (<0.01-1.54)	50 (89%)	3.88 (0.36-10.36)	0.004
Fusobacterium	18 (67%)	0.11 (<0.01-0.98)	39 (70%)	0.67 (<0.01-2.55)	0.170

**Actinobacteriota**	27 (100%)	5.28 (2.62-7.4)	56 (100%)	3.82 (2.58-6.92)	0.590
Corynebacterium **	26 (96%)	2.59 (0.83-4.75)	53 (95%)	0.55 (0.2-1.29)	<0.001
Alloscardovia **	19 (70%)	0.06 (<0.01-0.18)	42 (75%)	0.37 (0.01-1.92)	0.020
Varibaculum **	14 (52%)	0.01 (<0.01-0.13)	50 (89%)	0.48 (0.08-1.26)	<0.001
**Minor phyla**	25 (93%)	0.45 (0.3-1.25)	55 (98%)	0.78 (0.23-1.62)	0.970
Campylobacter **	22 (81%)	0.32 (0.06-0.6)	55 (98%)	0.65 (0.21-1.48)	0.012
Minor genera **	27 (100%)	11.63 (5.75-17.85)	56 (100%)	3.57 (1.6-6.32)	<0.001
Unclassified genera **	27 (100%)	4.29 (1.21-19.41)	56 (100%)	15.31 (8.1-20.02)	0.047

# n (%): number of samples in which the phylum/genus was detected (relative frequency of detection).

† Wilcoxon rank tests with Bonferroni correction.
