# Supplementary material for: Captive Breeding and Trichomonas gallinae Alter the Oral Microbiome of Bonelli’s Eagle Chicks
Source: Microb Ecol. 2022 Apr 7;85(4):1541–51. doi: 10.1007/s00248-022-02002-y (PMC10167124; doi:10.1007/s00248-022-02002-y)
Supplement: Supplementary file 6 — (PDF 124 KB) [file 248_2022_2002_MOESM6_ESM.pdf]

Supplementary Table S2. Relative frequencies, medians, and interquartile range (IQR) of the most abundant bacterial phyla (in bold) and genera detected in birds bred at nest with or without *Trichomonas* infection groups ordered by relative abundance.

	Nest Negative group		Nest Infected group		P-value†
**Phylum/Genus**	**n (%)[#]**	**Median (IQR)**	**n (%)[#]**	**Median (IQR)**	
**Firmicutes**	26 (100%)	62.06 (43.43-81.05)	30 (100%)	63.96 (51.11-75.01)	0.81
Megamonas	26 (100%)	39.19 (19.23-61.67)	30 (100%)	41.96 (26.24-52.86)	0.79
Peptostreptococcus	26 (100%)	5.69 (2.2-10.84)	30 (100%)	5.16 (3.64-7.93)	0.93
Gemella	24 (92%)	1.4 (0.48-3.29)	27 (90%)	1.05 (0.51-3.71)	0.94
Veillonella	25 (96%)	2.23 (1.56-2.88)	30 (100%)	2.54 (2-3.42)	0.13
Mycoplasma	25 (96%)	0.79 (0.41-1.92)	30 (100%)	1.02 (0.52-2.03)	0.42
Peptoniphilus	22 (85%)	0.25 (0.04-0.67)	28 (93%)	0.35 (0.06-0.52)	0.64
Staphylococcus	16 (62%)	0.01 (<0.01-0.05)	12 (40%)	<0.01 (<0.01-0.02)	0.18
**Bacteroidota**	26 (100%)	12.42 (5.38-20.01)	30 (100%)	14.61 (8.57-19.27)	0.29
Bacteroides	26 (100%)	9.92 (2.05-13.5)	30 (100%)	10.09 (7.43-15.63)	0.36
Ornithobacterium	24 (92%)	0.25 (0.14-0.64)	28 (93%)	0.24 (0.05-0.42)	0.33
**Fusobacteriota**	26 (100%)	6.08 (0.84-14.85)	30 (100%)	7.54 (2.2-14.02)	0.79
Oceanivirga	24 (92%)	4.54 (0.43-9.62)	26 (87%)	3.22 (0.39-9.81)	0.70
Fusobacterium	14 (54%)	0.11 (<0.01-1.76)	22 (73%)	1.07 (<0.01-3.1)	0.24
**Proteobacteria**	26 (100%)	4.57 (1.92-9.87)	30 (100%)	2.92 (2.24-6.95)	0.34
Suttonella	25 (96%)	0.43 (0.24-1.14)	29 (97%)	0.4 (0.13-1.06)	0.52
Psychrobacter	25 (96%)	0.29 (0.1-0.59)	23 (77%)	0.14 (0.03-0.57)	0.20
Sutterella	22 (85%)	0.42 (0.18-0.68)	29 (97%)	0.34 (0.22-0.59)	0.85
**Actinobacteriota**	26 (100%)	3.82 (2.95-5.24)	30 (100%)	3.78 (2.31-7.22)	0.95
Corynebacterium	26 (100%)	0.61 (0.26-2.27)	28 (93%)	0.5 (0.19-1.04)	0.30

Alloscardovia	20 (77%)	0.17 (0.03-0.75)	22 (73%)	0.61 (<0.01-2.76)	0.52
Varibaculum	22 (85%)	0.44 (0.07-1.08)	28 (93%)	0.56 (0.1-1.42)	0.48
**Minor phyla**	25 (96%)	0.93 (0.29-1.76)	30 (100%)	0.69 (0.22-1.22)	0.34
Campylobacter	25 (96%)	0.77 (0.23-1.67)	30 (100%)	0.62 (0.18-1.04)	0.38
Minor genera	26 (100%)	2.2 (1.06-3.31)	30 (100%)	2.11 (0.7-4.12)	0.65
Unclassified genera	26 (100%)	12.9 (8.78-17.4)	30 (100%)	17.31 (9.09-20.45)	0.22

[#] n (%): number of samples in which the phylum/genus was detected (relative frequency of detection).

† Wilcoxon rank tests with Bonferroni correction.
