# Supplementary material for: Captive Breeding and Trichomonas gallinae Alter the Oral Microbiome of Bonelli’s Eagle Chicks
Source: Microb Ecol. 2022 Apr 7;85(4):1541–51. doi: 10.1007/s00248-022-02002-y (PMC10167124; doi:10.1007/s00248-022-02002-y)
Supplement: Supplementary file 7 — (PDF 101 KB) [file 248_2022_2002_MOESM7_ESM.pdf]

Supplementary Table S3. Relative frequencies, medians, and interquartile range (IQR) of the most abundant bacterial phyla (in bold) and genera detected in birds bred at captivity with negative *T. gallinae* infection and *T. gallinae*-infected group ordered by relative abundance. **: Significant p values (lower than 0.05).

	Captivity Negative group		Captivity *T. gallinae*-Infected group		P-value†
**Phylum/Genus**	**n (%)#**	**Median (IQR)**	**n (%)#**	**Median (IQR)**	
**Firmicutes**	20 (100%)	49.46 (38.97-70.66)	7 (100%)	53.81 (43.48-65.84)	0.94
Enterococcus **	13 (65%)	1.22 (<0.01-11.28)	7 (100%)	23.85 (13.9-30.48)	0.01
Megamonas	19 (95%)	3.34 (0.45-17.76)	7 (100%)	2.04 (0.8-5.64)	0.50
Clostridium	13 (65%)	0.51 (<0.01-7.78)	6 (86%)	1.18 (0.26-7.35)	0.61
Lactobacillus	17 (85%)	0.53 (0.11-5.57)	7 (100%)	4.12 (2.13-7.37)	0.23
Staphylococcus **	16 (80%)	1.07 (0.02-2.93)	7 (100%)	7.24 (2.78-12.3)	0.03
Peptostreptococcus	18 (90%)	0.64 (0.09-4.00)	6 (86%)	0.7 (0.35-2.89)	1.00
Gemella	11 (55%)	0.10 (<0.01-2.65)	6 (86%)	0.11 (0.02-0.28)	0.69
Paeniclostridium	11 (55%)	0.02 (<0.01-0.33)	5 (71%)	0.05 (0.01-0.89)	0.44
Streptococcus	15 (75%)	0.39 (0.03-1.14)	7 (100%)	0.73 (0.48-2.3)	0.16
**Proteobacteria**	20 (100%)	22.63 (6.51-42.31)	7 (100%)	23.15 (16.48-40.26)	0.65
Escherichia-Shigella	12 (60%)	0.33 (<0.01-14.68)	7 (100%)	9 (3.62-35.67)	0.06
Psychrobacter	10 (50%)	<0.01 (<0.01-0.5)	2 (29%)	<0.01 (<0.01-0.02)	0.29
Proteus	11 (55%)	0.01 (<0.01-1.43)	6 (86%)	0.77 (0.39-2.32)	0.19
Devosia	5 (25%)	<0.01 (<0.01-0.01)	0 (0%)	<0.01 (<0.01-<0.01)	0.17
Klebsiella	2 (10%)	<0.01 (<0.01-<0.01)	2 (29%)	<0.01 (<0.01-4.9)	0.19
**Bacteroidota**	19 (95%)	4.47 (1.34-13.97)	7 (100%)	2.73 (2.1-4.05)	0.69
Bacteroides	18 (90%)	1.69 (0.50-9.57)	7 (100%)	1.1 (0.65-3.17)	0.64
**Actinobacteriota**	20 (100%)	5.86 (2.74-8.04)	7 (100%)	4.28 (2.18-5.82)	0.45

Corynebacterium	19 (95%)	3.01 (0.76-5.80)	7 (100%)	2.3 (1.28-3.25)	0.73
**Fusobacteriota**	18 (90%)	1.43 (0.16-4.79)	7 (100%)	0.47 (0.19-2.4)	0.80
Oceanivirga	13 (65%)	0.25 (<0.01-1.87)	6 (86%)	0.33 (0.14-0.36)	0.82
Fusobacterium	12 (60%)	0.09 (<0.01-0.89)	6 (86%)	0.13 (0.06-1.83)	0.32
Minor phyla	19 (95%)	0.51 (0.39-1.53)	6 (86%)	0.45 (0.2-0.86)	0.41
Minor genera	20 (100%)	9.70 (6.06-14.8)	7 (100%)	10.76 (3.43-13.45)	0.77
Unclassified genera	20 (100%)	10.15 (2.05-22.57)	7 (100%)	2.31 (1.17-3.65)	0.18

# n (%): number of samples in which the phylum/genus was detected (relative frequency of detection).

† Wilcoxon rank tests with Bonferroni correction.
