# Supplementary material for: Captive Breeding and Trichomonas gallinae Alter the Oral Microbiome of Bonelli’s Eagle Chicks
Source: Microb Ecol. 2022 Apr 7;85(4):1541–51. doi: 10.1007/s00248-022-02002-y (PMC10167124; doi:10.1007/s00248-022-02002-y)
Supplement: Supplementary file 8 — (PDF 108 KB) [file 248_2022_2002_MOESM8_ESM.pdf]

Supplementary Table S4. Relative frequencies, medians, and interquartile range (IQR) of the most abundant bacterial phyla (in bold) and genera detected in birds bred at nest with or without lesion ordered by relative abundance. **: Significant p values (lower than 0.05).

	Nest-No Lesion group		Nest-Lesion group		P-value†
**Phylum/Genus**	**n (%)#**	**Median (IQR)**	**n (%)#**	**Median (IQR)**	
**Firmicutes**	38 (100%)	65.03 (46.1-82.62)	18 (100%)	58.44 (44.79-72.74)	0.33
Megamonas	38 (100%)	43.13 (24.33-55.44)	18 (100%)	37.64 (19-49.98)	0.47
Peptostreptococcus	38 (100%)	6.12 (2.75-10.53)	18 (100%)	4.34 (2.94-7.45)	0.5
Gemella **	33 (87%)	0.99 (0.24-2)	18 (100%)	3.09 (1.07-4.78)	0.011
Veillonella	37 (97%)	2.44 (1.65-3.74)	18 (100%)	2.37 (2-2.85)	0.9
Mycoplasma	37 (97%)	0.86 (0.45-1.92)	18 (100%)	0.94 (0.53-2.03)	0.83
Peptoniphilus	34 (89%)	0.31 (0.06-0.52)	16 (89%)	0.27 (0.05-0.71)	0.96
Staphylococcus	20 (53%)	0.01 (<0.01-0.02)	8 (44%)	<0.01 (<0.01-0.02)	0.58
**Bacteroidota**	38 (100%)	12.42 (5.32-16.79)	18 (100%)	14.61 (10.68-24.79)	0.064
Bacteroides **	38 (100%)	7.89 (3.52-13.5)	18 (100%)	10.65 (8.81-18.25)	0.037
Ornithobacterium	35 (92%)	0.25 (0.07-0.64)	17 (94%)	0.24 (0.12-0.45)	0.67
**Fusobacteriota**	38 (100%)	5.29 (0.84-13.49)	18 (100%)	12.2 (5.54-16.14)	0.11
Oceanivirga	33 (87%)	2.41 (0.2-8.51)	17 (94%)	4.68 (1.78-14.09)	0.19
Fusobacterium	22 (58%)	0.33 (<0.01-1.96)	14 (78%)	1.23 (0.02-3.03)	0.23
**Proteobacteria**	38 (100%)	3.81 (1.84-14.25)	18 (100%)	3.12 (2.3-5.21)	0.61
Suttonella	37 (97%)	0.4 (0.18-1.18)	17 (94%)	0.46 (0.26-0.94)	0.81
Psychrobacter	33 (87%)	0.16 (0.04-0.59)	15 (83%)	0.19 (0.05-0.54)	0.83
Sutterella	34 (89%)	0.29 (0.17-0.66)	17 (94%)	0.41 (0.34-1.02)	0.11
**Actinobacteriota**	38 (100%)	3.72 (2.65-5.92)	18 (100%)	4.14 (2.4-7.01)	0.86

Corynebacterium	37 (97%)	0.61 (0.27-1.69)	17 (94%)	0.45 (0.18-1.02)	0.31
Alloscardovia	29 (76%)	0.17 (0.02-1.48)	13 (72%)	0.52 (0.02-2.23)	0.63
Varibaculum	33 (87%)	0.44 (0.1-1.08)	17 (94%)	0.73 (0.06-1.72)	0.5
**Minor phyla**	37 (97%)	0.61 (0.22-1.59)	18 (100%)	0.82 (0.4-1.22)	0.62
Campylobacter	37 (97%)	0.57 (0.2-1.5)	18 (100%)	0.7 (0.39-1.04)	0.61
Minor genera	38 (100%)	1.88 (0.7-3.96)	18 (100%)	2.42 (1.12-3.84)	0.95
Unclassified genera	38 (100%)	15.85 (7.83-20.37)	18 (100%)	14.16 (10.58-19.65)	0.79

[#] n (%): number of samples in which the phylum/genus was detected (relative frequency of detection).

† Wilcoxon rank tests with Bonferroni correction.
